# Apathy following Bilateral Deep Brain Stimulation of Subthalamic Nucleus in Parkinson's Disease: A Meta-Analysis

**DOI:** 10.1155/2018/9756468

**Published:** 2018-05-21

**Authors:** Ying Wang, Yongsheng Li, Xiaona Zhang, Anmu Xie

**Affiliations:** ^1^Department of Neurology, The Affiliated Hospital of Qingdao University, Qingdao, China; ^2^Department of Neurology, The Ninth People Hospital of Qingdao City, Qingdao, China

## Abstract

Bilateral deep brain stimulation of subthalamic nucleus (STN-DBS) has proven effective in improving motor symptoms in Parkinson's disease (PD) patients. However, psychiatric changes after surgery are controversial. In this study, we specifically analyzed apathy following bilateral STN-DBS in PD patients using a meta-analysis. Relevant articles utilized for this study were obtained through literature search on PubMed, ScienceDirect, and Embase databases. The articles included were those contained both pre- and postsurgery apathy data acquired using the Starkstein Apathy Scale or Apathy Evaluation Scale with patient follow-up of at least three months. A total of 9 out of 86 articles were included in our study through this strict screening process. Standardized mean difference (SMD), that is, Cohen's d, with a 95% confidence interval (CI) was calculated to show the change. We found a significant difference between the presurgery stage and the postsurgery stage scores (SMD = 0.35, 95% CI: 0.17∼0.52, *P* < 0.001). STN-DBS seems to relatively worsen the condition of apathy, which may result from both the surgery target (subthalamic nucleus) and the reduction of dopaminergic medication. Further studies should focus on the exact mechanisms of possible postoperative apathy in the future.

## 1. Introduction

Parkinson's disease (PD) is the second most common neurodegenerative disease after Alzheimer's disease and is characterized by bradykinesia, rigidity, resting tremors, and postural instability [1]. In addition to these motor symptoms, PD patients also suffer from many nonmotor symptoms including mood and behavior disorders, cognitive changes, autonomic system-failure, sensory symptoms, and sleep disturbances [2–4]. Following long-term treatment using antiparkinsonian medications, the presence of dyskinesia and symptom fluctuations becomes a major therapeutic challenge. Thus, deep brain stimulation (DBS) has recently become a preferable surgical therapy to treat PD. The globus pallidus internus (GPi) and the subthalamic nucleus (STN) are the main targets of the stimulating loci [5]. Neurosurgeons implant the electrodes using an approach that combines intraoperative recording and stimulation. The targets are identified using preoperative magnetic resonance imaging and intraoperative electrophysiological recordings [6].

It has been well established that bilateral deep brain stimulation of subthalamic nucleus (STN-DBS) significantly improves the primary motor symptoms as well as some nonmotor symptoms, such as sensory symptoms and sleep disturbances [7, 8]. However, apathy, a common mood disorder in PD patients after bilateral STN-DBS, is controversial. Apathy has been described as a quantitative reduction in purposeful behaviors and self-generated voluntary actions [9], which cannot be attributed to any impairment of consciousness or any emotional or cognitive disorder [10]. Apathy is also known to significantly increase burden on caregivers and has negative effects on treatment and long-term outcome [11, 12].

Many studies have reported increases in apathy after STN-DBS [13–18], while others show opposite outcomes [19–21]. Neurologists cannot forecast this behavioral outcome when advising surgery to their patients and patients' family. Therefore, we performed this quantitative meta-analysis with strict inclusion criteria to study the effect of bilateral STN-DBS on apathy and expected to draw a conclusion and provide useful reference for clinical practice.

## 2. Materials and Methods

### 2.1. Search Strategy

Literature searches of the PubMed, ScienceDirect, and Embase databases up to January 2017 were performed to identify relevant articles published in English. The search terms were (“bilateral deep brain stimulation” OR “bilateral subthalamic stimulation”) OR (bilateral stimulation AND “subthalamic nucleus”) AND (“Parkinson disease” OR “Parkinson's disease”) AND “apathy”. In addition, we searched the references of the identified studies to find other satisfactory studies. This task was completed by two reviewers independently. When disagreements arose, a third reviewer was consulted.

### 2.2. Inclusion and Exclusion Criteria

The inclusion criteria were the following: (1) Full-text publications written in English, (2) At least 10 patients in the study, (3) The patients were followed up for at least 3 months, (4) presurgery and postsurgery apathy data obtained through the Starkstein Apathy Scale or Apathy Evaluation Scale (The Starkstein Apathy Scale consists of 14 questions and was designed specifically for patients with PD. Scores range from 0 (least severe apathy) to 42 (most severe apathy). A score of 14 or greater indicates clinically significant apathy [22]. The Apathy Evaluation Scale contains 18 questions with scores ranging from 18 to 72, and a higher score is associated with a worse condition [23].), (5) The data were analyzed in the form of mean and standard deviation, (6) The missing data could be obtained using definite methods written in the Cochrane handbook [24].

The exclusion criteria were (1) reviews, meta-analysis, book chapters, letters to the editor, or case reports with no original data, (2) duplicate reports with identical data, (3) data from nonhuman species, and (4) insufficient original data.

### 2.3. Quality Assessment

Two reviewers evaluated the quality of the studies using the Methodological Index for Non-randomized Studies (MINORS). The MINORS covers 8 different areas, and each area is scored 0(not reported), 1 (reported but inadequate), or 2 (reported and adequate). A score greater than 10 indicates a good quality study [25].

### 2.4. Extraction

The data were extracted from the selected articles by two researchers independently, while differences were resolved by consulting a third reviewer. The following information was extracted: first author's name, year of publication, sample size, patient characteristics, time of following up, DBS programming, the state (on/off) in the postoperative evaluation, and the relevant presurgery and postsurgery apathy data.

### 2.5. Statistical Analysis

We combined the results of each article using standard meta-analytic methods to estimate the overall efficacy, tolerability, and safety of STN-DBS. STATA statistics software (Version 12.0, Stata Corporation, College Station, Texas 77,845 USA) was used to analyze available data. The data collected on apathy using the Starkstein Apathy Scale or the Apathy Evaluation Scale were considered continuous data. Since there were two scales used in our study, an estimate of the combined effect sizes utilizing standardized mean difference (SMD), that is, Cohen's d, was given, with a 95% confidence interval (CI). SMD, a standard statistic, was used to show the comparisons of presurgery and postsurgery change. This value reflects an intervention-induced change of the outcome on an average and is used as a summary statistic in meta-analysis when the studies were measured in different ways [26]. The Q-test and *I*^2^-statistics were used to evaluate the degree of heterogeneity between studies. The fixed-effects model was employed if *I*^2^ < 50%; otherwise, the random-effects model was used [27]. Sensitivity analysis was performed by excluding each study and reanalyzing the remaining studies. Begg's test, which measures funnel plot asymmetry, was used to assess publication biases. A value of <0.05 for Begg's test was considered statistically significant publication bias [28].

## 3. Results

### 3.1. Characteristics of Eligible Studies

**Figure 1:**
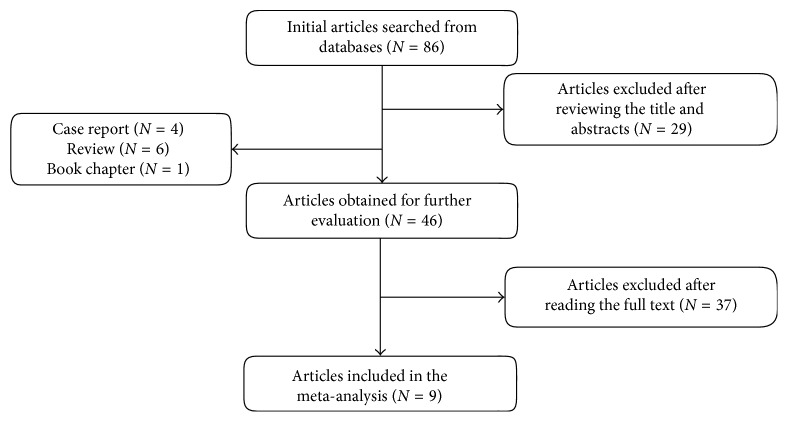
Flow chart of eligible articles.

Overall, 86 articles were initially retrieved. After reviewing titles and abstracts, 29 articles, 4 case reports, 6 reviews, and 1 book chapter were excluded. After reading the full-texts of the remaining articles, 9 studies met all of our inclusion criteria and were picked up for this meta-analysis.  Figure 1 shows the flow chart of the screening process.

All the included studies were follow-up type studies, with following up time ranging from 3 months to 17 months. The sample size was 253, and 111 patients (44%) were assessed using the Starkstein Apathy Scale, the others using the Apathy Evaluation Scale. All PD patients involved underwent bilateral STN-DBS and were evaluated in the state of drug on and drug on/stimuli on before and after surgery. The main areas studied are described in Table 1.

Table 2 shows the results from 9 included articles evaluated using MINORS analyses on 8 different areas. All studies had clearly stated aims, prospective collections of data, endpoints appropriate to the aim of the study, and follow-up periods appropriate to the aim of the study. Although not all trials had inclusion of consecutive patients and unbiased assessments of the study endpoint, the total scores show a good quality of each study.

### 3.2. Quantitative Synthesis

**Figure 2:**
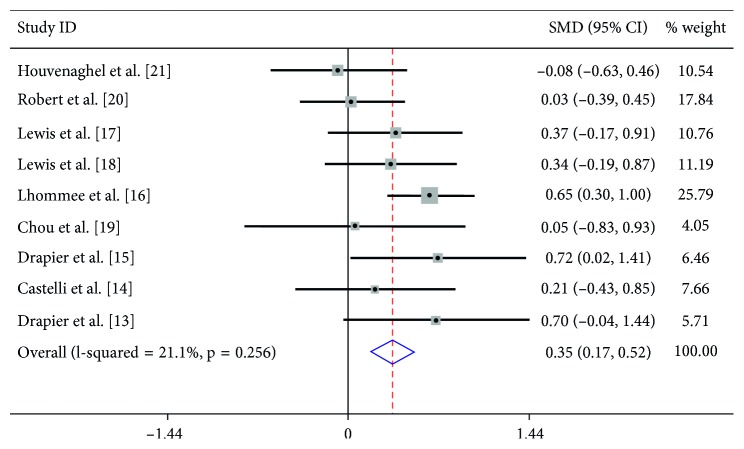
Forest plot for the change in apathy observed presurgery and postsurgery.

The heterogeneity between the included studies showed that *I*^2^ = 21.1%; therefore, the fixed-effects model was used to count the pooled SMD. Based on the comparison of preoperative and postoperative change, we found that there was a significant difference in the score between the presurgery stage and the postsurgery stage (SMD = 0.35, 95% CI: 0.17∼0.52, *P* < 0.001) (Figure 2). Further subgroup analysis showed that follow-up did not have an effect on the condition of apathy (*p*=0.256).

**Figure 3:**
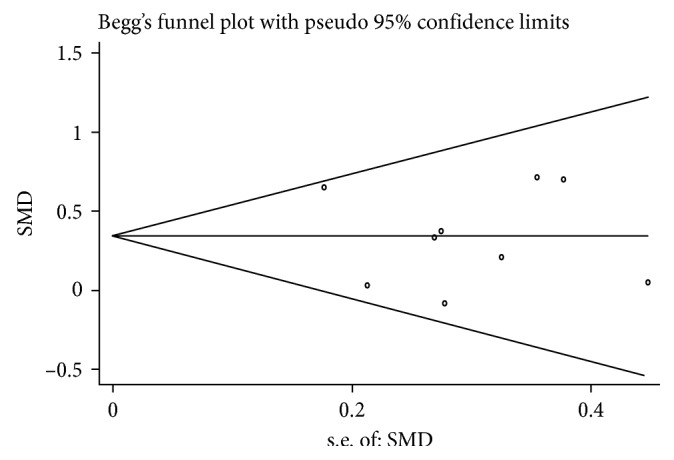
Funnel plot for publication bias in selection of studies.

In the sensitivity analysis, each study was omitted by turns to show the influence of every article contributing to this meta-analysis. No significant alterations were found in the pooled SMD, which showed a high level of stability of this meta-analysis. Begg's test was used to assess publication bias, and the funnel plot was approximately symmetric, indicating that there was no publication bias(Figure 3).

## 4. Discussion

In recent years, bilateral STN-DBS has been performed widely in order to treat advanced PD patients. STN-DBS involves the application of electrical stimuli, with specific pulse amplitude, duration, and frequency to produce a functional lesion within the subthalamic nucleus [29]. Compared to the conventional pharmacotherapy, it can afford to decrease motor fluctuations, reduce “off” time, and show improvement in dyskinesia [30]. There are several meta-analyses examining the postoperative condition of PD patients. Tan et al. and Xie et al. reported that STN-DBS could improve Unified Parkinson's disease rating scale III (UPDRS-III) scores and quality of life (QOL) and allow recovery of verbal fluency [31, 32]. Many published meta-analyses have showed evidence for an adverse effect on cognition, depression and anxiety [33–35]. The present article is, to our knowledge, the first meta-analysis focusing on the effects of DBS on apathy.

Apathy is defined as a lack of motivation characterized by diminished goal-oriented behavior and cognition and reduced emotional expression [36]. The prevalence of apathy in PD varies from 17% to 70% depending on the sample populations, diagnostic criteria, and evaluation tools utilized [7]. PD caregivers live with a burden resulting from the apathy condition of patients, similar to the caregivers of other neurological disorders. Apathy also has negative effects on treatment and long-term outcome. Neurologists should carefully consider the target of choice for PD patients who are eligible for DBS as a means to overcome the adverse effect of long-term treatment of antiparkinsonian medication [11, 12]. Specifically, attention should be paid to the change in apathy following bilateral STN-DBS in PD as it has implications for treatment and care. The apathetic scales we used in this study are the Starkstein Apathy Scale and the Apathy Evaluation Scale: the former was designed specifically for PD patients and the latter is regarded as the most psychometrically robust apathy scale [37].

The present meta-analysis included 9 studies containing 253 PD patients comparing the differences in apathy between presurgery and postsurgery patients. Through strict methodological and statistical analysis, our data suggested that there was a statistical significant difference in the scores between the presurgery stage and the postsurgery stage (SMD = 0.35, 95% CI: 0.17∼0.52, *P* < 0.001), which means that bilateral STN-DBS did seem to worsen the PD patients' apathetic condition. However, the subgroup analysis of the relationship between follow-up and the change in apathy score failed to support this conclusion (*p*=0.256).

We were not able to draw a conclusion about the clinical significance of the finding. There are several limitations of this article. First, the studies included were all follow-up studies, not randomized controlled trails with control groups, which hindered us from analyzing whether the progression of PD played a role in the change of apathy, nor do levodopa equivalent daily dose (LEDD) or other confounding factors. Second, due to the limited sample size, the power that was used to detect a true difference between presurgery and postsurgery may be not strong. Additionally there were only a few studies in the subgroup analysis resulting in a low statistical power when analyzing the effect of follow-up on the condition of apathy.

STN-DBS seemed to worsen the condition of apathy regardless of the follow-up, and we attempted to unravel the reasons why some articles reported that apathy scores in PD were worsened after bilateral STN-DBS. The exact mechanisms of changes to apathy after surgery remain unclear.

Successful STN-DBS is accompanied by a decrease of dopaminergic medication at all times resulting from improvement of patients' motor symptoms, which suggests that a dopaminergic deficit may be an explanation for the pathogenesis of some forms of apathy [38]. Thobois et al. exposited that early postoperative apathy corresponds to a dopaminergic abstinence syndrome caused by a postoperative reduction in dopaminergic medication which discloses presynaptic degeneration of mesolimbic dopaminergic terminals [39]. Czernecki et al. performed a trial with ropinirole, a selective dopaminergic agonist (DA), showing that the reduction of dopaminergic medication may induce postoperative apathy [40]; however, the study had a small sample size. In another study, researchers found addition of DAs in the patients who suffered from more severe apathy after STN-DBS might lead to confusion rather than improvement [41]. Accounting for this, Carriere et al. wrote in their article that there were PD patients with either dopaminergic apathy (related to dopaminergic limbic denervation) or dopa-resistant apathy (related to striatal limbic atrophy), the latter of which may be related to more extensive spread of the disease [42].

Researchers did not make a conclusion about the exact relationship between post-DBS apathy and reduction of dopaminergic medication after surgery. More studies pay attention to the operation targets to explain the apathic condition after STN-DBS. The STN is described to play an important role in each of the five corticobasal ganglia-thalamocortical circuits, each of which have specific motor, oculomotor, associative, or limbic functions [43]. There are three functional domains of STN: sensorimotor (dorsolateral), limbic (medial), and cognitive-associative (ventromedial) [44, 45]. Drapier et al. has reported that apathetic patients after surgery are stimulated more ventrally and internally in STN, as opposed to the nonapathetic patients who are stimulated closer to the dorsolateral area [13]. For other surgery targets, previous studies provided contrary outcomes in regards to the change between presurgery and postsurgery scores. Lozachmeur et al. found there was no significant difference between presurgery and postsurgery assessments for apathy when they chose GPi to be the target [46]. However, many studies have found that the STN-DBS is superior at reducing the LEDD compared to GPi-DBS [47–49]. The smaller reduction of dopaminergic medication after GPi-DBS may weaken the worse score after surgery when compared to the condition of STN-DBS. As mentioned above, we can speculate that both the surgery target (subthalamic nucleus) and the reduction of dopaminergic medication are involved in the apathetic condition after STN-DBS.

In conclusion, the condition of apathy seems to be worsened following bilateral STN-DBS in PD. Further studies should focus on the exact mechanisms of apathy following bilateral STN-DBS. Considering the limitations mentioned above, further studies with more specific information and larger sample sizes should be carried out, and caution should be taken in interpreting our findings.

## Figures and Tables

**Table 1:** Characteristics of the eligible studies.

Number	Author	*N*	Age	Disease duration	DBS programming	State in the evaluation	Scale	Follow-up	Preoperative score	Preoperative LEDD	Postoperative score	Postoperative LEDD
1	Houvenaghel et al. [21]	26	56.6 ± 7.4	11.47 ± 4.54	Bilateral STN-DBS	Drug on and stimuli on	AES	3 months	31.8 ± 7.0	1271.2 ± 555.6	31.2 ± 7.7	758.0 ± 407.79
2	Robert et al. [20]	44	56.3 ± 7.5	11.4 ± 4.1	Bilateral STN-DBS	Drug on and stimuli on	AES	3 months	31.4 ± 6.4	1280.8 ± 632.4	31.6 ± 7.1	889.9 ± 209.3
3	Lewis et al. [17]	27	61.1 ± 9.1	12.7 ± 6.7	Bilateral STN-DBS	Drug on and stimuli on	AES	1 year	34.04 ± 9.58	831.5 ± 425.91	37.44 ± 8.71	359.23 ± 264.46
4	Lewis et al. [18]	28	61.1 ± 8.9	12.43 ± 6.74	Bilateral STN-DBS	Drug on and stimuli on	AES	1 year	33.85 ± 9.71	832 ± 426	37.0 ± 8.91	359.3 ± 264.5
5	Lhommee et al. [16]	67	57.8 ± 7.2	10.5 ± 3.1	Bilateral STN-DBS	Drug on and stimuli on	SAS	1 year	6.2 ± 3.5	1026 ± 459	9.4 ± 4.5	284 ± 312
6	Chou et al. [19]	10	62.1 ± 6.5	9.1 ± 5.8	Bilateral STN-DBS	Drug on and stimuli on	SAS	6 months	13.2 ± 8.6	1164.9 ± 752.9	13.6 ± 7.4	567.9 ± 512.4
7	Drapier et al. [15]	17	56.9 ± 8.7	11.8 ± 2.6	Bilateral STN-DBS	Drug on and stimuli on	AES	3 months	37.2 ± 5.5	-	42.5 ± 8.9	-
8	Castelli et al. [14]	19	62.1 ± 4.2	14.7 ± 5.0	Bilateral STN-DBS	Drug on and stimuli on	SAS	17 months	11.6 ± 4.1	1192.5 ± 415.7	12.6 ± 5.3	571.6 ± 274.8
9	Drapier et al. [13]	15	59.7 ± 7.6	12.2 ± 2.8	Bilateral STN-DBS	Drug on and stimuli on	SAS	6 months	13.0 ± 6.5	1448 ± 400	18.8 ± 9.7	1127 ± 482

AES: Apathy Evaluation Scale; SAS: Starkstein Apathy Scale.

**Table 2:** MINORS scores of eligible studies.

Number	A	B	C	D	E	F	G	H	Total
1 [21]	2	0	2	2	0	2	2	1	11
2 [20]	2	2	2	2	0	2	2	1	13
3 [17]	2	0	2	2	0	2	1	1	10
4 [18]	2	0	2	2	0	2	2	0	10
5 [16]	2	0	2	2	0	2	2	1	11
6 [19]	2	0	2	2	0	2	2	1	11
7 [15]	2	0	2	2	0	2	2	1	11
8 [14]	2	2	2	2	0	2	2	0	12
9 [13]	2	0	2	2	0	2	2	0	10

A: a clearly stated aim; B: inclusion of consecutive patients; C: prospective collection of data; D: endpoints appropriate to the aim of the study; E: unbiased assessment of the study endpoint; F: follow-up period appropriate to the aim of the study; G: loss to follow-up less than 5%; H: prospective calculation of the sample size.
